# Activities of Daily Living Object Dataset: Advancing Assistive Robotic Manipulation with a Tailored Dataset

**DOI:** 10.3390/s24237566

**Published:** 2024-11-27

**Authors:** Md Tanzil Shahria, Mohammad H. Rahman

**Affiliations:** 1 Computer Science, University of Wisconsin-Milwaukee, Milwaukee, WI 53211, USA; rahmanmh@uwm.edu; 2 Mechanical Engineering, University of Wisconsin-Milwaukee, Milwaukee, WI 53211, USA

**Keywords:** image dataset, activities of daily living, assistive robotics, object detection, deep learning

## Abstract

The increasing number of individuals with disabilities—over 61 million adults in the United States alone—underscores the urgent need for technologies that enhance autonomy and independence. Among these individuals, millions rely on wheelchairs and often require assistance from another person with activities of daily living (ADLs), such as eating, grooming, and dressing. Wheelchair-mounted assistive robotic arms offer a promising solution to enhance independence, but their complex control interfaces can be challenging for users. Automating control through deep learning-based object detection models presents a viable pathway to simplify operation, yet progress is impeded by the absence of specialized datasets tailored for ADL objects suitable for robotic manipulation in home environments. To bridge this gap, we present a novel ADL object dataset explicitly designed for training deep learning models in assistive robotic applications. We curated over 112,000 high-quality images from four major open-source datasets—COCO, Open Images, LVIS, and Roboflow Universe—focusing on objects pertinent to daily living tasks. Annotations were standardized to the YOLO Darknet format, and data quality was enhanced through a rigorous filtering process involving a pre-trained YOLOv5x model and manual validation. Our dataset provides a valuable resource that facilitates the development of more effective and user-friendly semi-autonomous control systems for assistive robots. By offering a focused collection of ADL-related objects, we aim to advance assistive technologies that empower individuals with mobility impairments, addressing a pressing societal need and laying the foundation for future innovations in human–robot interaction within home settings.

## 1. Introduction

The global demographic shift towards an aging population and the rising prevalence of disabilities have intensified the need for advanced assistive technologies that promote autonomy and improve the quality of life for individuals with mobility impairments. In the United States alone, over 61 million adults live with a disability [1], representing approximately 26% of the adult population. Mobility disabilities are among the most common, affecting millions who rely on wheelchairs or other assistive devices for daily movement. Moreover, almost one-third of mobility device users need assistance from another person in one or more of the activities of daily living (ADLs), such as eating, grooming, dressing, and personal hygiene [2]. Dependence on caregivers for these fundamental activities can impact the independence and psychological well-being of individuals with disabilities.

Assistive robotic technologies, particularly wheelchair-mounted robotic arms, have emerged as promising tools to enhance the independence of individuals with mobility impairments. These robotic systems are designed to assist users in performing ADLs by extending their physical capabilities and enabling them to interact with objects in their environment. However, the practical deployment of such assistive robots faces challenges, primarily due to the complexity of control interfaces and the cognitive load required for manual operation. For many users, especially those with severe disabilities, controlling a multi-degree-of-freedom robotic arm can be daunting and may not lead to the desired level of independence.

Automation of robotic control through advanced perception systems offers a viable solution to these challenges. Deep learning-based object detection models have revolutionized computer vision [3], enabling robots to perceive and interpret complex environments with remarkable accuracy. By integrating such models, assistive robots can autonomously identify, locate, and manipulate objects relevant to ADLs, thereby reducing the operational burden on users. However, the success of these models is heavily dependent on the availability of large, high-quality datasets that accurately represent the target domain [4].

Existing large-scale image datasets, such as the Common Objects in Context (COCO) dataset [5], Open Images [6], the Large Vocabulary Instance Segmentation (LVIS) dataset [7], and collections from platforms like Roboflow Universe [8], have been instrumental in advancing object detection and segmentation algorithms. These datasets provide extensive collections of labeled images, covering a wide variety of objects and scenes. Nonetheless, they are general-purpose datasets not specifically tailored for assistive robotics applications. They often include a broad spectrum of objects, many of which are irrelevant or unsuitable for manipulation by assistive robots in home environments. Additionally, these datasets contain mislabeled or low-quality images, which can adversely affect the training and performance of deep learning models in real-world scenarios [9].

The lack of specialized datasets focusing on manipulable ADL objects presents a significant barrier to the development of effective perception systems for assistive robots. Domain-specific datasets have been shown to improve model performance in specialized applications by providing relevant contextual information and reducing the model’s exposure to irrelevant data. For assistive robotics, a dataset that captures the diversity of objects encountered in daily living tasks, annotated with high precision, is essential for training models that can operate reliably in home settings. In this study, we aim to address these limitations by creating a specialized, high-quality dataset tailored for ADL object detection in assistive robotics. The primary objectives of this research are:

- Dataset curation and compilation: to curate and compile a comprehensive dataset of manipulable ADL objects from existing large-scale datasets, focusing on items relevant to assistive robots in home environments.
- Annotation standardization: to standardize the annotations of the collected images into a uniform YOLO (You Only Look Once) Darknet annotation format [10], facilitating seamless integration with state-of-the-art object detection models.
- Data quality enhancement: to filter and enhance the dataset by removing mislabeled and low-quality images through a combination of automated filtering using a pre-trained YOLOv5x [11] model and manual review processes.

Our contributions include the development of a novel dataset of ADL objects specifically curated for assistive robotics applications, which we will make publicly available as an open-source resource. This dataset addresses the critical need for high-quality, task-specific data that can significantly improve the performance of object detection models in assistive scenarios. By providing a focused collection of manipulable objects commonly encountered in daily living tasks, we aim to bridge the data gap in this domain and facilitate advancements in assistive technologies.

This work addresses a pressing societal need and lays the foundation for future innovations in human–robot interaction within home settings. By enhancing the perceptual capabilities of assistive robots, we can empower individuals with mobility impairments to achieve greater independence and improve their overall quality of life. The results of this study underscore the importance of targeted datasets in driving advancements in deep learning and robotics, highlighting the potential for specialized data to unlock new levels of performance and reliability in assistive technologies.

## 2. Related Work

The advancement of object detection has been significantly propelled by the development of large-scale, annotated datasets that serve as benchmarks for training and evaluating deep learning models. The Common Objects in Context (COCO) dataset [5] is one of the most influential, providing over 200,000 images with detailed annotations for object detection, segmentation, and captioning tasks. COCO’s diverse range of everyday objects in natural contexts has made it a cornerstone for research in computer vision. Similarly, the Open Images dataset [6] offers an extensive collection of approximately 9 million images annotated with image-level labels, bounding boxes, and visual relationships, encompassing a vast array of object classes. The Large Vocabulary Instance Segmentation (LVIS) dataset [7] extends these efforts by focusing on instance segmentation with a large vocabulary of over 1000 object categories, aiming to address the long-tail distribution of object frequencies in the real world.

While these general-purpose datasets have been instrumental in advancing object detection techniques, their applicability to robotic manipulation—especially in assistive robotics—is limited. The objects included often do not represent the manipulable items commonly encountered in activities of daily living (ADLs), and the images may not reflect the specific environmental contexts of home settings where assistive robots operate. Also, the annotations in these datasets are not tailored to the needs of robotic perception systems, which require precise and relevant information for object manipulation tasks.

Recognizing the need for specialized datasets, several efforts have been made to create collections more suited to robotic applications. The Yale-CMU-Berkeley (YCB) Object and Model Set [12] is a notable example, providing physical objects along with high-resolution 3D scans for benchmarking robotic manipulation algorithms. This dataset includes everyday items like kitchenware and tools selected for their relevance to manipulation tasks and variability in shape, size, and texture. The Washington RGB-D Object dataset [13] offers RGB-D images of household objects from multiple viewpoints, facilitating research in object recognition and pose estimation using depth information, which is valuable for robotic grasping and manipulation.

In the context of robotic grasping, the Cornell Grasping dataset [14] provides images annotated with positive and negative grasp rectangles, aiding the development of algorithms for detecting graspable regions on objects. While these datasets have advanced specific areas of robotic manipulation, they often lack the breadth and diversity needed for comprehensive ADL object detection in assistive robotics. They may focus on a limited set of objects or specific tasks like grasp detection without encompassing the full range of items and scenarios encountered in daily living environments.

Assistive robotics has seen growing interest, with research focusing on developing robotic systems that aid individuals with disabilities in performing ADLs. Smarr et al. [15] explored robotic assistance in dressing, utilizing computer vision to detect clothing items and plan manipulation strategies. This study highlights the potential of assistive robots to enhance independence and underscore the challenges posed by complex control interfaces and the need for reliable perception systems.

Deep learning techniques have been increasingly adopted in assistive robotics to enhance autonomy and adaptability. For instance, Shahria et al. [16] developed a vision-based assistive robot capable of recognizing and manipulating objects for picking up an object task, employing the YOLOv5 [11] model for object detection. However, these approaches often rely on general-purpose datasets or limited custom datasets that do not fully capture the diversity of objects and contexts relevant to assistive tasks in home environments. This reliance can lead to performance issues when models encounter unfamiliar objects or settings not represented in their training data.

The limitations of existing datasets in addressing the specific needs of assistive robotics have been acknowledged in recent studies. Ruiz-del-Solar et al. [17] emphasized the importance of domain-specific datasets for improving the performance of robotic perception systems in assistive applications. They noted that datasets tailored to the objects and environments typical of home settings could significantly enhance the accuracy and reliability of object detection models used in assistive robots.

To address these challenges, some researchers have created specialized datasets focusing on specific aspects of assistive robotics. For example, the Assistive Grasping dataset [18] includes a collection of objects commonly found in household environments, annotated for grasp planning tasks. While valuable, these datasets are often limited in scope and do not provide the comprehensive coverage needed for general ADL object detection.

Our work seeks to fill this gap by creating a large-scale, high-quality dataset specifically curated for manipulable ADL objects in home environments. By systematically collecting and annotating images from existing large-scale datasets—focusing on objects pertinent to daily living tasks—we aim to provide a resource that addresses the specific challenges faced in assistive robotics. This contribution is intended to facilitate the development of advanced perception systems that can empower individuals with disabilities through more autonomous and effective assistive technologies.

## 3. Dataset Creation

The development of a specialized dataset tailored for ADL object detection in assistive robotics necessitates meticulous data collection and rigorous quality assurance processes. This section outlines the methodology employed in creating our dataset, comprising two primary phases: data collection and data filtering. The goal was to assemble a high-quality dataset of manipulable ADL objects with precise annotations, suitable for training deep learning models in assistive robotic applications.

### 3.1. Data Collection

The initial step involved identifying a comprehensive list of ADL objects relevant to assistive robotic manipulation in home environments. Building upon our previous study [19], which identified approximately 90 objects that assistive robots need to interact with to provide effective assistance, we curated a focused subset of 54 ADL objects for this study.

The previous study [19] was conducted under a National Institute on Disability, Independent Living, and Rehabilitation Research (NIDILRR)-funded project. A multidisciplinary team comprising experts in assistive technology, occupational therapy, and human–robot interaction collaboratively evaluated the initial list. Through expert review and iterative feedback from end-users and caregivers, we ensured that the selected objects are both relevant and practical for enhancing user independence. The refinement from 90 to 54 objects was focused on objects that are autonomously or semi-autonomously manipulable by assistive robots and essential for performing ADLs, considering factors such as size, shape, and frequency of use in daily tasks. The selected objects encompass a range of items commonly encountered in daily activities. This targeted selection ensures that the dataset is directly applicable to the contexts in which assistive robots operate.

To compile the dataset, we sourced images from four major open-source datasets known for their extensive collections and diversity:

- COCO dataset: We downloaded over 80,000 images covering 24 classes from the COCO dataset. COCO is renowned for its high-quality annotations and variety of everyday objects in context, making it a valuable resource for our purposes.
- Open Images dataset: From the Open Images dataset, we acquired over 31,000 images encompassing 39 classes. This dataset provides a vast collection of images with rich annotations, including object bounding boxes and class labels.
- LVIS dataset: The LVIS dataset contributed over 27,000 images across 36 classes. LVIS focuses on a large vocabulary of object categories and instance segmentation, offering detailed annotations suitable for object detection tasks.
- Roboflow Universe: For classes not sufficiently represented in the aforementioned datasets, we searched the Roboflow Universe platform, obtaining over 19,000 images spanning 14 classes. Roboflow Universe aggregates datasets from various contributors, providing access to specialized datasets that complement our requirements.

After collecting the images, we standardized all annotations by converting them to the YOLO Darknet format. This involved transforming existing annotation formats into a unified structure specifying object class labels and bounding box coordinates relative to image dimensions. Class IDs were mapped according to our predefined list of 54 objects, ensuring consistency across the dataset. Uniformity in annotation format and class labeling is critical for effective model training and broader adoption by the research community. The list of 54 object classes is presented in Table 1.

In this study, we focused on curating and filtering existing image data to create a high-quality dataset tailored for assistive robotic applications. We did not alter or augment the original images; instead, we meticulously selected images based on the specific needs of our application. By leveraging existing datasets and applying rigorous filtering criteria, we ensured that the dataset is both relevant and of high quality without introducing any modified or artificially generated content.

### 3.2. Data Sources and Ethical Considerations

All images and annotations obtained from the mentioned datasets are released under the Creative Commons Attribution 4.0 International License (CC BY 4.0), which permits the sharing and adaptation of the material in any medium or format for any purpose, even commercially, provided that appropriate credit is given, a link to the license is provided, and any changes made are indicated. This license explicitly allows for the redistribution of the images and annotations, enabling us to legally incorporate and redistribute these data within our newly created dataset. In compliance with the license terms, we have provided appropriate attribution to the original creators of each dataset, included license information in our documentation, and indicated any modifications performed. By adhering to these requirements, we ensure that our dataset respects the legal and ethical standards set forth by the original data providers, facilitating its use and redistribution by the broader research community and fostering advancements in assistive robotics.

### 3.3. Data Filtering

Ensuring the quality and reliability of the dataset is paramount for training effective deep-learning models. To this end, we implemented a two-phase filtering process comprising automated filtering and manual verification. This rigorous approach was designed to eliminate mislabeled data, incorrect annotations, and low-quality images that could adversely affect model performance.

#### 3.3.1. Automated Filtering

The automated filtering phase utilized a pre-trained YOLOv5x [11] model, known for its high accuracy and efficiency in object detection tasks. The selection of YOLOv5x was based on several key considerations:

- Balance of speed and accuracy: YOLOv5x offers a superior balance between detection speed and accuracy, making it well-suited for processing large volumes of images efficiently without compromising on performance. This balance is crucial for maintaining the feasibility of the automated filtering process within our resource constraints.
- Proven effectiveness on the COCO dataset: Because YOLOv5x is trained on the COCO dataset, it demonstrates proficiency in detecting a wide range of common objects that overlap significantly with our selected ADL classes. This pre-existing competence reduces the likelihood of false negatives and enhances the reliability of the automated filtering process.
- Ease of integration and customization: YOLOv5x is highly adaptable and can be easily fine-tuned to specific datasets. Its compatibility with popular deep learning frameworks and availability of extensive documentation facilitated seamless integration into our data processing pipeline.
- Community and support: YOLOv5x benefits from a strong community and active support, providing access to numerous resources, updates, and best practices that informed our implementation strategy.

While alternative object detection models such as Faster R-CNN, EfficientDet, and Mask R-CNN offer distinct architectural advantages, the scope of our study prioritized efficiency and practicality in the data filtering phase. Conducting comprehensive comparisons with these models would require significant additional computational resources and time, which were beyond the constraints of our current project. Also, YOLOv5x’s established performance on datasets similar to ours provided a reliable foundation for initial data quality assurance. However, we acknowledge that exploring other models could provide valuable comparative insights and may be considered in future work to further validate and potentially enhance the filtering process.

Automated filtering process: The automated filtering process involved the following steps:

- Model inference: for each image, the YOLOv5x model was used to perform object detection, generating predictions that include detected classes, bounding box coordinates, and confidence scores.
- Annotation comparison: the model’s predictions were compared against the annotations in our dataset. If the predicted classes matched the annotated classes, and if the confidence scores exceeded a predefined threshold (set at 0.5), the image and its annotations were considered valid and retained.
- Mismatch identification: images where the model’s predictions did not align with the annotations were flagged as potentially mislabeled or poorly annotated. These images were moved to a separate directory for discarding.
- Unknown classes handling: for annotated classes not recognized by the pre-trained model (due to the model’s limited class set), images were set aside for manual verification to prevent the inadvertent removal of valid data.

The utilization of a pre-trained YOLOv5x model in automated filtering offers several advantages:

- Efficiency: automated filtering accelerates the initial data cleansing process, enabling rapid processing of a large volume of images.
- Consistency: the model applies uniform validation criteria across all images, reducing human error and bias.
- Quality assurance: filtering out images with incorrect annotations or mislabeled objects enhances the overall dataset quality, which is crucial for training reliable models.

The automated filtering process is exemplified by a Python script. The script systematically loads images and annotations, performs model inference, and categorizes images based on the comparison results, ensuring that only high-quality data are retained.

#### 3.3.2. Manual Verification

Following automated filtering, manual verification was conducted to refine the dataset further. This phase involved human reviewers using a Python script to visualize images with annotations, allowing for meticulous inspection of annotation accuracy and image quality. Criteria for excluding images during manual verification included:

- Incorrect labels: annotations where the class labels did not correspond to the objects present in the image.
- Poorly labeled images: inaccurate bounding boxes, missing annotations, or annotations that only partially covered the object.
- Annotation inconsistencies: discrepancies between the number of objects present and the number of annotations (e.g., an image with n objects but n+m or n-m annotations).
- Incorrect bounding box sizes: bounding boxes that were disproportionately large or small relative to the object could impair the model’s ability to learn precise localization.

**Figure 1:**
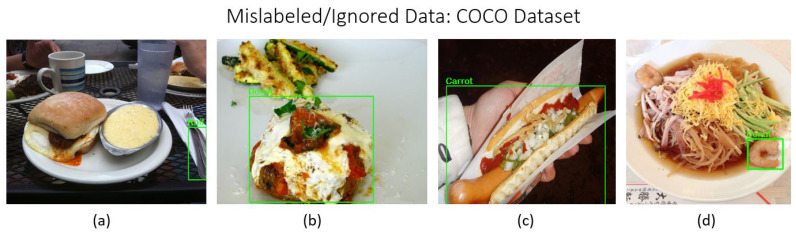
Examples of mislabeled or ignored data from the COCO dataset: (**a**) incorrect class label; (**b**) incorrect class label; (**c**) incorrect class label; (**d**) incorrect class label.

**Figure 2:**
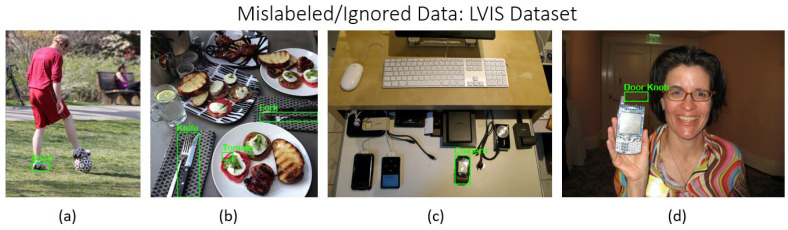
Examples of mislabeled or ignored data from the LVIS dataset: (**a**) missing annotation for one class; (**b**) missing annotations for multiple classes; (**c**) incorrect class label; (**d**) incorrect bounding box information.

**Figure 3:**
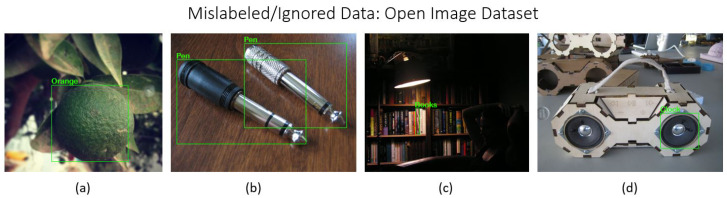
Examples of mislabeled or ignored data from the Open Images dataset: (**a**) incorrect class label; (**b**) incorrect class label; (**c**) missing annotations; (**d**) incorrect class label.

Images meeting quality standards were retained, while those failing to comply were removed. Special consideration was given to images containing multiple objects. If an image contained *n* instances of object *A* and *m* instances of object *B*, and the annotations accurately reflected all instances of at least one object class, the image was retained for that class. The images in Figure 1, Figure 2 and Figure 3 illustrate examples of mislabeled or ignored data excluded during the filtering process from three major datasets: COCO, LVIS, and Open Images. These examples highlight instances where data quality issues, such as incorrect annotations or irrelevant object labels and annotation missing for multiple objects, were identified and subsequently filtered out to ensure the integrity and accuracy of the final dataset.

#### 3.3.3. Error Analysis and Class Imbalance

Error analysis: during the automated filtering and manual verification processes, we identified several common types of data issues within the source datasets. The most frequent issues included:

1. Incomplete annotations for multiple objects: Many images contained multiple instances of the same object class, but annotations were provided for only a single instance. This resulted in underrepresented object instances within the dataset.
2. Inaccurate bounding box information: A significant number of annotations had bounding boxes that were either too large, too small, or improperly aligned with the actual object boundaries, leading to imprecise localization.
3. Incorrect class labels: Mislabeling of object classes was prevalent, where the annotated class did not correspond to the object present in the image. This included both overgeneralized and overly specific class labels.
4. Poor image quality: Low-resolution images, excessive noise, and poor lighting conditions were common, affecting the clarity and detectability of objects.

Class imbalance: Addressing class imbalance is crucial for training models that perform uniformly across all object classes. In our dataset, certain classes were significantly underrepresented compared with others. To mitigate this, we primarily focused on the following strategies:

- Selective data collection: We prioritized collecting additional images for underrepresented classes from the Roboflow Universe platform and other supplementary sources to balance the class distribution.
- No data augmentation: Importantly, we did not apply data augmentation techniques, such as oversampling or the use of generative adversarial networks (GANs), to generate synthetic data. This decision was made to preserve the authenticity and integrity of the data, ensuring that all samples represent real-world instances without introducing potential biases or artifacts that synthetic data might introduce.

### 3.4. Dataset Integrity and Scope

It is important to note that our dataset is not intended to supplant existing open-source datasets nor to suggest that they lack quality. Instead, we aim to provide a specialized dataset specifically designed for ADL object manipulation in assistive robotics. By focusing on objects pertinent to daily living tasks and ensuring high annotation quality through cross-verification, our dataset addresses a niche yet critical area in the field.

The final dataset comprises a curated collection of high-quality images with accurate annotations, ready for training deep learning models to enhance the perceptual capabilities of assistive robots. Table 2 presents the object count statistics from various image sources, comparing downloaded and filtered datasets across all sources. This provides a comprehensive overview of the data distribution for each object category.

## 4. ADL Dataset

The final dataset resulting from our meticulous data collection and rigorous filtering processes is a comprehensive resource tailored specifically for assistive robotic manipulation in activities of daily living (ADLs). This section provides an in-depth overview of the dataset’s characteristics, including the distribution of images across object classes, the diversity of sources, and the quality enhancements achieved through our filtering methods.

### 4.1. Dataset Composition

Our dataset encompasses 112,925 images after filtering, covering 54 ADL object classes crucial for assistive robots operating in home environments. The images were sourced from four prominent open-source datasets: COCO, Open Images, LVIS, and Roboflow Universe. The diversity of these sources ensures a wide range of visual contexts, object appearances, and environmental variations, enhancing the robustness of models trained on this dataset.

To facilitate effective training and validation, the total dataset was randomly split into two directories: train and valid. Approximately 85% of the data is kept in the train directory for model training, while the remaining 15% is stored in the valid directory for validation purposes. This random splitting strategy helps ensure that both sets are representative of the overall data distribution, contributing to the development of models with strong generalization capabilities.

#### Class Distribution

The dataset includes objects commonly encountered in daily tasks, such as utensils, personal care items, electronic devices, and household goods. Table 3 provides a comprehensive summary of the class-wise distribution of images and instances across the proposed dataset, including detailed counts for each class as well as aggregate totals for the number of images and instances processed. The class distribution is balanced to the extent possible, with particular attention paid to objects of high relevance to assistive tasks. Some key observations from the dataset include:

- High-frequency classes: Objects like Cup, Bottle, and Hat have a higher number of images, reflecting their frequent use and importance in daily activities. For instance, the Cup class comprises a total of 9760 images after filtering, sourced primarily from COCO and Open Images.
- Specialized classes: Less common but essential objects, such as Pill Bottle, Door Knob, and Milk Gallon, are included to cover a comprehensive range of assistive needs. While smaller in image count, these classes are critical for developing models that can handle specialized tasks.
- Diverse representations: By integrating data from multiple sources, the dataset captures variations in object appearance due to different angles, lighting conditions, backgrounds, and occlusions, essential for training robust models capable of generalizing to real-world scenarios.

### 4.2. Data Quality Enhancements

The filtering processes significantly improved the dataset’s quality by removing mislabeled, poorly annotated, or low-quality images. The overall reduction in the number of images from the initial download to the final dataset underscores the stringency of our quality assurance measures.

- Automated filtering impact: The automated filtering phase eliminated images with incorrect or inconsistent annotations, as identified by discrepancies between model predictions and annotations. For example, after filtering, the Bottle class was reduced from 8501 images to 6967 images from the COCO dataset.
- Manual verification impact: Manual inspection further refined the dataset, ensuring that annotations precisely matched the objects in the images. This step was crucial for classes where automated filtering might overlook nuanced errors, particularly in images with multiple objects or complex scenes.

### 4.3. Data Source Contributions

The contributions of each data source to the final dataset are as follows:

- COCO dataset: This dataset provided the majority of images, with 69,956 images across various classes after filtering. COCO’s high-quality annotations and contextual diversity make it a valuable foundation for the dataset.
- Open Images dataset: This dataset contributed 25,488 images after filtering. Open Images enriched the dataset with additional classes and varied visual representations.
- LVIS dataset: This dataset added 13,993 images post-filtering. LVIS’s focus on a large vocabulary of objects enhanced the diversity of object categories.
- Roboflow Universe: This dataset supplied 3488 images after filtering, particularly for classes underrepresented in other datasets. This inclusion ensured coverage of specialized objects necessary for assistive tasks.

### 4.4. Dataset Accessibility and Usage

The dataset is publicly available (under CC BY 4.0) as an open-source resource to encourage collaboration and advancement in the field. Researchers and developers can leverage this dataset to train and evaluate object detection models specifically tailored for ADL tasks, accelerating the development of semi-autonomous control systems for assistive robots. The dataset can be accessed/download from the following link: https://doi.org/10.6084/m9.figshare.27263424 (accessed on 24 November 2024).

## 5. Experimental Evaluation

To assess the effectiveness of our ADL dataset, we conducted experiments using the YOLOv8n deep learning model. This evaluation aimed to demonstrate the dataset’s capability to enhance object detection performance in assistive robotics applications. Also, we performed a comparative analysis with models trained on existing public datasets to highlight the advantages and unique characteristics of our ADL dataset.

### 5.1. Model Validation and Performance Evaluation

#### 5.1.1. Experimental Setup

We selected YOLOv8n for its optimal balance between accuracy and computational efficiency, which is crucial for real-time applications in assistive robotics. The model was fine-tuned on our ADL dataset for 50 epochs using a learning rate of 0.01, a batch size of 32, and an input image size of 640 × 640 pixels. Training was conducted on a system equipped with an NVIDIA RTX A2000 GPU (manufactured by NVIDIA Corporation, headquartered in Santa Clara, CA, USA) to ensure efficient processing.

#### 5.1.2. Evaluation Metrics

The model was evaluated using standard object detection metrics to provide a comprehensive understanding of its performance:

- Mean average precision (mAP@0.5): measures the average precision across all classes at an intersection over union (IoU) threshold of 0.5.
- Mean average precision (mAP@0.5:0.95): averages the mAP over multiple IoU thresholds ranging from 0.5 to 0.95 in increments of 0.05.
- Precision: the ratio of true positive detections to the total number of positive detections.
- Recall: the ratio of true positive detections to the total number of actual positives in the dataset.

#### 5.1.3. Results

Table 4 presents the performance metrics of the fine-tuned YOLOv8n model on the validation set of our ADL dataset. The model achieved a mAP@0.5 of 0.592 and a mAP@0.5:0.95 of 0.458. Additionally, the precision and recall metrics of the model are 0.572 and 0.608, respectively.

#### 5.1.4. Inference Speed

To ensure that the fine-tuned model remains suitable for real-time applications in assistive robotics, we evaluated its inference speed. The average inference times per image are presented in Table 5. The inference times remained consistent before and after fine-tuning, with the YOLOv8n model averaging 18.45 ms post-fine-tuning compared with 18.10 ms pre-fine-tuning. This consistency confirms that the enhanced performance does not incur additional computational overhead, maintaining the model’s suitability for real-time robotic operations.

#### 5.1.5. Discussion

The performance of the model demonstrates the effectiveness of our ADL dataset in enhancing model performance for assistive robotics applications. Fine-tuning allows the YOLOv8n model to learn specific features and contexts relevant to ADL objects, which are not adequately represented in general-purpose datasets. The maintenance of real-time inference speeds ensures that these enhancements are practical for deployment in assistive robotic systems.

### 5.2. Comparative Analysis with Existing Public Datasets

To demonstrate the advantages and unique characteristics of our ADL dataset in assistive robotics applications, we conducted a comparative analysis with existing public datasets commonly used for object detection tasks, such as COCO and Open Images. This analysis focuses on the relevance of the datasets to assistive robotics, coverage of ADL-related objects, annotation quality, and their suitability for training models in assistive applications.

#### 5.2.1. Relevance to Assistive Robotics

Our ADL dataset is specifically curated to include 54 object classes that are directly relevant to activities of daily living and suitable for manipulation by assistive robots in home environments. In contrast, while the COCO and Open Images datasets contain a wide variety of object classes, they are general purpose and not tailored to the needs of assistive robotics. Many ADL-relevant objects are underrepresented or absent in these datasets, limiting their effectiveness for training models intended for assistive applications.

As illustrated in Table 6, our dataset offers comprehensive coverage of ADL-relevant objects, whereas COCO and Open Images include only a subset of these classes. This extensive coverage ensures that models trained on our dataset are exposed to a broader range of objects pertinent to assistive robotics, thereby enhancing their applicability and performance in real-world assistive tasks.

#### 5.2.2. Annotation Quality and Consistency

Our dataset emphasizes high-quality annotations, achieved through rigorous filtering and manual verification processes detailed in Section 3.3. This ensures accurate bounding boxes and consistent class labels, which are critical for the precision required in assistive robotics. High-quality annotations contribute to better model performance and reliability, essential factors for deployment in real-world assistive applications.

In contrast, existing datasets like COCO and Open Images, while extensive, may contain mislabeled data or inconsistent annotations, particularly for objects that are less common or have ambiguous definitions. Such issues can adversely affect the performance of models in critical applications like assistive robotics, where accuracy and reliability are paramount. By providing meticulously curated and annotated data, our ADL dataset mitigates these challenges, offering a more dependable foundation for training robust object detection models tailored to assistive robotics.

## 6. Discussion

The creation of the specialized ADL dataset presented in this study addresses a critical gap in assistive robotics, particularly in enhancing the autonomy and effectiveness of assistive robots. Integrating deep learning-based object detection models into assistive robots can revolutionize how individuals with mobility impairments interact with their environment, reducing dependence on caregivers and improving quality of life. This discussion elaborates on how our dataset supplements existing work, the technical advancements it enables, and its implications for future research and development.

### 6.1. Contribution to the Field

Our dataset supplements existing large-scale datasets like COCO, Open Images, and LVIS by providing a focused collection of images specifically tailored for ADL object detection in assistive robotics. While these general-purpose datasets have been invaluable for advancing computer vision algorithms, they lack the specificity required for assistive applications. By curating a dataset with 54 manipulable ADL objects, we ensure that the training data align closely with the operational environment of assistive robots. This alignment is crucial for developing models that perform reliably in home settings, where the variability of objects and contexts can significantly affect model performance.

Also, the rigorous filtering and annotation processes enhance the dataset’s quality, addressing common issues in existing datasets such as mislabeled data and inconsistent annotations. High-quality annotations are essential for training deep learning models that require precise localization and classification capabilities, particularly when assistive robots must manipulate objects safely and effectively.

Enhancement of robotic perception and control systems: Beyond the development of a specialized dataset, our work significantly contributes to the advancement of robotic perception and control systems in assistive robotics. By providing high-quality, task-specific data, we enable the training of more accurate and reliable object detection models tailored to the unique demands of assistive tasks. This leads to improved autonomy and efficiency in robotic manipulation, reducing the cognitive and operational burdens on users. Additionally, our dataset serves as a benchmark for evaluating and comparing different deep learning architectures in the context of assistive robotics, fostering further research and innovation in this domain. The standardized annotation format and rigorous quality assurance processes we established can be adopted by future studies, promoting consistency and reproducibility in assistive robotics research.

### 6.2. Technical Advancements

The availability of this specialized dataset enables several technical advancements in assistive robotics. Models trained on the dataset can achieve higher accuracy in detecting and localizing objects used in ADLs as they are exposed to various instances and contexts specific to assistive tasks, reducing the domain gap and improving generalization. Accurate object detection is crucial for effective robotic manipulation as it enables perception systems to handle complex scenes, recognize objects in cluttered environments, and adapt to variations in appearance. Additionally, automating object detection reduces cognitive load on users, allowing them to issue high-level commands instead of managing intricate robotic movements. The dataset provides a benchmark for evaluating object detection models, encouraging innovation and standardization in assistive robotics.

### 6.3. Tangible Outcomes and Validation

The deployment of our ADL dataset is expected to yield significant tangible outcomes in the realm of assistive robotics, particularly in enhancing the autonomy and effectiveness of robotic systems in supporting activities of daily living. By enabling the development of more accurate and reliable object detection models, our dataset facilitates autonomous robotic manipulation of essential ADL objects, thereby increasing user independence and reducing reliance on human caregivers. The anticipated benefits include improved quality of life for individuals with mobility impairments, greater economic efficiency through the reduction of caregiving costs, and accelerated innovation in assistive technologies. To confirm that these benefits meet expectations, we will establish a robust scientific validation framework that combines empirical performance evaluations, user studies, and longitudinal assessments. This framework will involve quantitative metrics such as mean average precision (mAP), precision, and recall to objectively measure model performance and qualitative feedback from end-users and caregivers to assess usability and real-world impact. Also, real-world deployment tests with assistive robotic systems will be conducted to observe and validate the practical effectiveness of the trained models in dynamic home environments. Through this comprehensive validation approach, we aim to ensure that the deployment of our dataset delivers the intended benefits and contributes meaningfully to the advancement of assistive robotics.

### 6.4. Limitations and Future Work

Despite the significant contributions of our dataset, some limitations present opportunities for future research:

1. Class imbalance: While efforts were made to balance the dataset, some classes have fewer images due to data availability in source datasets. This imbalance could affect model performance on underrepresented classes. Future work may involve collecting additional data or employing data augmentation techniques to address this issue.
2. Limited viewpoint diversity: The current dataset may not fully capture objects from multiple angles and backgrounds, which is important for simulating complex real-world environments. Future work will involve incorporating images of objects from various perspectives and settings to enhance model robustness against variations in object appearance.
3. Dynamic interactions: Considering the actual usage scenarios of assistive robots, the dataset lacks images of objects in use or in motion, such as door handles at various stages of operation. Including such dynamic representations in future datasets could improve the models’ ability to handle real-world manipulation tasks involving moving objects.
4. Three-dimensional perception: Assistive robots often rely on depth information for accurate manipulation. Integrating RGB-D data or 3D models could improve object detection and localization, facilitating more precise interactions.
5. Incorporation of time series data: Future research will focus on extending the dataset to include time series data, capturing dynamic interactions and temporal variations. This enhancement aims to better simulate real-world environments where objects may be in motion or subject to changing conditions, thereby improving the models’ ability to handle dynamic manipulation tasks.
6. Interactive learning and user feedback mechanisms: We plan to explore the application of the dataset in interactive learning frameworks for robots. Implementing user feedback mechanisms will allow assistive robots to optimize model performance in real time, adapting to individual user preferences and specific contextual requirements. This approach aims to enhance the adaptability and responsiveness of assistive robotic systems, ensuring they meet the diverse needs of users in dynamic settings.

## 7. Conclusions

This study addressed a critical gap in assistive robotics by creating a specialized dataset of manipulable activity of daily living (ADL) objects tailored for assistive robots. Recognizing the limitations of existing general-purpose datasets, we curated a focused collection encompassing 54 essential ADL objects. We ensured high-quality annotations and image integrity suitable for training deep learning models through meticulous data collection and rigorous filtering—both automated using a pre-trained YOLOv5x model and manual verification. Our dataset stands as a significant contribution to the field, providing a valuable resource that enhances the perceptual capabilities of assistive robots. By enabling more accurate object detection and localization, it facilitates the development of semi-autonomous control systems that reduce user cognitive load and increase independence for individuals with mobility impairments. The dataset’s alignment with real-world assistive tasks ensures that models trained on it are better equipped to operate reliably in home environments.

The implications of this work extend beyond immediate applications, laying the groundwork for future research in human–robot interaction, contextual understanding, and personalized assistive technologies. By making the dataset publicly available, we invite the research community to build upon our efforts, fostering innovation that can lead to more advanced, user-friendly assistive robots. In conclusion, our specialized ADL dataset fills a crucial void in assistive robotics and opens avenues for significant advancements in deep learning applications within this domain. It represents a step forward in empowering individuals with disabilities, contributing to enhanced autonomy and improved quality of life.

## Figures and Tables

**Table 1:** The list of 54 object classes along with their class id.

0: Apple	1: Handbag	2: Fork	3: Carrot	4: Donut	5: Backpack
6: Books	7: Cellphone/Mobile	8: Bottles	9: Remote	10: Pizza	11: Keyboard
12: Mouse	13: Knife	14: Glasses	15: Cup	16: Spoon	17: Bowl
18: Banana	19: Orange	20: Broccoli	21: Hot Dog	22: Clock	23: Laptop
24: Eraser	25: Pencil Box	26: Notebook	27: Pen	28: Ruler	29: Sharpener
30: Lipstick	31: Sock	32: Table Lamp	33: Pill Bottle	34: Lotion Bottle	35: Hat
36: Food Can	37: Wallet	38: Perfume	39: Camera	40: Shoe	41: Toilet Tissue
42: Scissor	43: Highlighter	44: Plum	45: Avocado	46: Peach	47: Tomato
48: Key	49: Bag of chips	50: Wrist Watch	51: Door Knob	52: Milk Gallon	53: Pencil

**Table 2:** Object count statistics from various image sources (downloaded and filtered data).

ID	Object Name	Downloaded Data				Filtered Data				Total
		COCO	Open Image	LVIS	Roboflow	COCO	Open Image	LVIS	Roboflow	
0	Apple	1586	389	1207		1191	376	717		2284
1	Handbag	6841	652	1859		4253	216	1107		5576
2	Fork	3555		1861		2602		1177		3779
3	Carrot	1683	499	1222		1370	441	777		2588
4	Donut	1523				1365				1365
5	Backpack	5528	165	1905		3345	90	1188		4623
6	Books	5332	1791	1903		3283	938	1004		5225
7	Cellphone	4803	1778		80	3477	1486		48	5011
8	Bottles	8501	3808			6967	3567			10,534
9	Remote	3078				2491				2491
10	Pizza	3166	588	1881		2908	547	1407		4862
11	Keyboard	2115	1299			1627	739			2366
12	Mouse	1876	512			1671	438			2109
13	Knife	4326	537	1868		2928	324	1170		4422
14	Glass	2533				2233				2233
15	Cup	9189	1117	1521		7698	1055	1007		9760
16	Spoon	3529	383	1127		2207	331	682		3220
17	Bowl	7111	689	1922		5355	455	1244		7054
18	Banana	2243	589	1787		1974	536	1344		3854
19	Orange	1699	883			1324	797			2121
20	Broccoli	1939	210	1309		1627	201	970		2798
21	Hot Dog	1222	72			997	64			1061
22	Clock	4659	884	1844		3840	830	1457		6127
23	Laptop	3524	5123		79	3245	4788		42	8075
24	Eraser		18	318			6	126		132
25	Pencil Box				258				110	110
26	Notebook		189	310			30	72		102
27	Pen		536	339	36		415	60	10	485
28	Ruler		36		540		30		107	137
29	Sharpener				846				120	120
30	Lipstick				190				94	94
31	Sock		231	1945	758		120	12	62	194
32	Table lamp				946				220	220
33	Pill Bottle				188				93	93
34	Lotion Bottle				651				194	194
35	Hat		6292	1932		5223	347			5570
36	Food Can		445	1051			83	70		153
37	Wallet		113	1041			38	127		165
38	Perfume		13	930			7	190		197
39	Camera		1721	1391		1178	111			1289
40	Shoe		1915	1237			25	218		243
41	Toilet Tissue		156		931		121		284	405
42	Scissors		172	707	829		170	497	10	677
43	Highlighter		128	494			11	264		275
44	Plum			1089				165		165
45	Avocado		117	1723			3	102		105
46	Peach		99	71	655		42	2	78	122
47	Tomato		1213	1470			18	111		129
48	Keys		195	941			24	165		189
49	Bag of Chips			575				213		213
50	Wrist Watch			1089				240		240
51	Door Knob			1710				590		590
52	Milk Gallon			224				47		47
53	Pencil		153	475			19	169		188

**Table 3:** Class-wise statistics of the final dataset, including the number of images and instances for each class.

Class ID	Number of Images	Total Instances	Class ID	Number of Images	Total Instances
0	2284	6176	27	485	607
1	5576	9937	28	137	165
2	3779	5343	29	120	143
3	2588	8438	30	94	188
4	1365	6789	31	194	600
5	4623	7132	32	220	242
6	5225	18,384	33	93	106
7	5011	6977	34	194	273
8	10,534	28,694	35	5570	6990
9	2491	4934	36	153	155
10	4862	7828	37	165	184
11	2366	3172	38	197	198
12	2109	2541	39	1289	1409
13	4422	6995	40	243	442
14	2233	7255	41	405	465
15	9760	20,414	42	677	709
16	3220	5283	43	275	275
17	7054	14,059	44	165	543
18	3854	10,768	45	105	143
19	2121	10,728	46	122	149
20	2798	7896	47	129	164
21	1061	2669	48	189	301
22	6127	7980	49	213	261
23	8075	9941	50	240	288
24	132	178	51	590	590
25	110	110	52	47	155
26	102	126	53	188	276
**Summary**					
Total number of images with only one class				110,202	
Total number of images with more than one class				2723	
Total number of images in the dataset				112,925	
Total number of instances in the dataset				236,768	

**Table 4:** Performance metrics of fine-tuned models.

Metric	YOLOv8n (Fine-Tuned)
mAP@0.5	0.592
mAP@0.5:0.95	0.458
Precision	0.572
Recall	0.608

**Table 5:** Average inference times (milliseconds per image) of YOLOv8n models.

Model	Inference Time (ms)
Fine-tuned YOLOv8n	18.45
Pre-trained YOLOv8n	18.10

**Table 6:** Comparison of ADL-relevant object classes across datasets.

Dataset	Total Classes	ADL-Relevant Classes
ADL dataset	54	54
COCO dataset	80	24
Open Images	600	39

## Data Availability

Our dataset is released under the Creative Commons Attribution 4.0 International (CC BY 4.0) license. This license allows for sharing, adaptation, and distribution of the dataset, provided appropriate credit is given to the original creators. The dataset can be accessed from the following link: https://doi.org/10.6084/m9.figshare.27263424 (accessed on 24 November 2024). Full license terms are available at https://creativecommons.org/licenses/by/4.0/ (accessed on 24 November 2024).

## Abbreviations

The following abbreviations are used in this manuscript:

ADLs	Activities of daily living
COCO	Common Objects in Context
LVIS	Large Vocabulary Instance Segmentation
YOLO	You Only Look Once
YCB	Yale-CMU-Berkeley
RGB-D	Red, green, and blue plus depth
